# Quality Issues of Laboratory Reagents

**DOI:** 10.4103/0973-6247.42698

**Published:** 2008-07

**Authors:** MANOJ A. KAHAR

**Affiliations:** Blood Bank, Indian Red Cross Society, Navsari, Gujarat, India. E-mail: manoj_kahar@yahoo.com

Dear Sir,

I send my sincere thanks to you and your team members for coming out with a very good Asian Journal of Transfusion Science. I have gone through each and every line of all the issues published till date and I have found them to be extremely useful. You and Your team members deserve special thanks because for Blood Bankers like me working in resource poor sectors, subscribing and reading costly international journals on transfusion medicine and blood banking is a dream to be fulfilled.

AJTS has been a BOON to people like me working in charitable institutes which do not have funds for journals. I have few queries related to blood banking techniques and procedures. I would be highly obliged if you can answer them

1) We know that Monoclonal Anti D (IgG + IgM) is preferred over Monoclonal Anti D (only IgM) to pick up weaker variants of D antigen, then what is the role of Monoclonal Anti D (only IgM) and why do companies prepare and market them?

2) Acceptable quality of Anti Human Globulin reagent as per quality parameters detailed in NABH and DGHS publications have some technical issues to be used in routine practice

a) Agglutination of red cells sensitized with anti D serum containing not more than 0.2 mg/ml antibody. How to measure this 0.2 mg/ml antibody activity in the anti D serum vial?

b) Agglutination of red cells sensitized with a complement binding antibody (e.g. anti Le a) How to prepare or get such sensitized cells?

c) Agglutination of red cells coated with C3b and C3d and no/weak agglutination with C4 coated cells How to prepare or get such red cells coated with C3b and C3d and also C4 coated cells?

